# RIS-Aided Proactive Mobile Network Downlink Interference Suppression: A Deep Reinforcement Learning Approach

**DOI:** 10.3390/s23146550

**Published:** 2023-07-20

**Authors:** Yingze Wang, Mengying Sun, Qimei Cui, Kwang-Cheng Chen, Yaxin Liao

**Affiliations:** 1 National Engineering Laboratory for Mobile Network Technologies, Beijing University of Posts and Telecommunications, Beijing 100876, China; wang_ying_ze1993@bupt.edu.cn (Y.W.); smy_bupt@bupt.edu.cn (M.S.); liaoyaxin@bupt.edu.cn (Y.L.); 2 Department of Electrical Engineering, University of South Florida, Tampa, FL 33620, USA; kwangcheng@usf.edu

**Keywords:** proactive mobile network (PMN), reconfigurable intelligent surface (RIS), asynchronous advantage actor–critic (A3C), interference suppression, reinforcement learning (RL)

## Abstract

A proactive mobile network (PMN) is a novel architecture enabling extremely low-latency communication. This architecture employs an open-loop transmission mode that prohibits all real-time control feedback processes and employs virtual cell technology to allocate resources non-exclusively to users. However, such a design also results in significant potential user interference and worsens the communication’s reliability. In this paper, we propose introducing multi-reconfigurable intelligent surface (RIS) technology into the downlink process of the PMN to increase the network’s capacity against interference. Since the PMN environment is complex and time varying and accurate channel state information cannot be acquired in real time, it is challenging to manage RISs to service the PMN effectively. We begin by formulating an optimization problem for RIS phase shifts and reflection coefficients. Furthermore, motivated by recent developments in deep reinforcement learning (DRL), we propose an asynchronous advantage actor–critic (A3C)-based method for solving the problem by appropriately designing the action space, state space, and reward function. Simulation results indicate that deploying RISs within a region can significantly facilitate interference suppression. The proposed A3C-based scheme can achieve a higher capacity than baseline schemes and approach the upper limit as the number of RISs increases.

## 1. Introduction

Recent advancements in state-of-the-art applications, including intelligent manufacturing, autonomous driving, and remote operations, have necessitated that mobile networks support communication with an exceptionally low latency [1,2,3]. This demand has prompted the emergence of ultra-reliable low-latency communication (URLLC) as one of the three communication scenarios within fifth generation mobile networks (5G). Despite significant efforts in recent years to reduce communication latency in 5G closed-loop architectures, these approaches often involve excessive control overhead messages, leading to unacceptable latency [4]. To address this issue, researchers are exploring methods to integrate perception, computation, and communication within mobile network architectures. This novel approach replaces conventional direct interaction control methods with historical data mining and perception of the surrounding environment to obtain relevant and necessary information. Consequently, a proactive mobile network (PMN) architecture is proposed [5,6,7]. The PMN architecture is considered to have significant theoretical value and holds the potential for deployment in future 6G networks [8,9,10].

**Figure 1:**
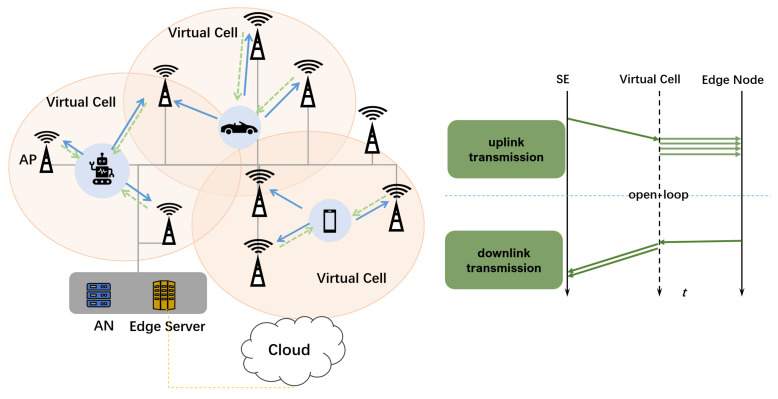
Network infrastructure framework for a PMN: access points (APs) offer spatial coverage and signal backhaul, while the anchor node (AN) is responsible for executing advanced operations and the edge server provides data processing in proactive mobile communication. The smart machine (SM) associates proactively with the APs to construct a virtual cell. Data are transmitted immediately upon generation, regardless of uplink or downlink, without extra control single exchange.

A PMN employs an open-loop transmission approach and utilizes a virtual cell architecture to achieve low-latency communication [11,12]. As depicted in Figure 1, the radio access network (RAN) comprises access points (APs) governed by an anchor node (AN). The AN is responsible for executing advanced networking capabilities and predictive network management in collaboration with the edge server. Multiple APs within the AN’s coverage area work together with a focus on machine centricity to establish a virtual cell, ensuring an uninterrupted service. In this architecture, data transmission occurs promptly upon generation, employing open-loop transmission for both the uplink and the downlink without additional control information exchange [13,14,15]. By leveraging environment perception sensors and powerful edge computing capabilities, the PMN eliminates the need for retransmission and acknowledgment processes. Compared to the data transmission process in 5G or other classic closed-loop networks, the PMN eradicates control links associated with a single data transmission, such as waiting for latency and post-transmission compensation, to an extreme extent. This compression results in the air interface latency performance condensing to a single one-way transmit time, thereby achieving extremely low-latency communication.

While the PMN holds the potential to achieve minimal communication latency, ensuring transmission-reliable capacity presents a significant challenge. Traditional approaches relying on interactive control protocols conflict with the PMN’s requirement to avoid real-time direct closed-loop control. Furthermore, the PMN does not conduct real-time resource allocation for individual transmission duties. Additionally, the channel resources available to different smart machines (SMs) are not independent, which limits efficiency within a restricted frequency bandwidth. This situation is further exacerbated by the impact of virtual cell technology, which introduces substantial inter–user interference and compounds the difficulty of ensuring reliable capacity in the PMN [6,16].

To address these challenges, reconfigurable intelligent surfaces (RISs) offer a potential solution. By manipulating the phase shifts of reflecting elements, RISs have emerged as a promising technology for configuring the wireless environment [17]. Multiple RISs can be strategically deployed within the PMN’s coverage area under the control of the AN. By judiciously modulating the RISs, the signal of interest can be amplified via direct refraction while co-channel interference is suppressed. However, the effective utilization of RISs necessitates precise real-time channel information, which proves challenging to obtain within the PMN due to the absence of real-time feedback associated with transmission.

In this paper, we propose an RIS-assisted interference suppression scheme based on the asynchronous advantage actor–critic (A3C) algorithm to surmount this challenging problem, which combines deep reinforcement learning (DRL) to control the RISs dynamically. Regarding the system design, we consider the fairness of the use of the network by SMs within the region and construct the goal as a max-min channel capacity problem. Our approach enables adaptive adjustments of the RISs without relying on accurate real-time feedback, maximizing the target in downlink transmission. Through experimental evaluations and simulations, the proposed A3C-based RIS-assisted scheme demonstrates its capability to effectively mitigate interference, enhance transmission reliability, and optimize the overall network performance.

The main contributions of this paper are as follows:

- We propose introducing RIS technology to solve the extensive and severe inter-user interference problem in PMN downlink communication. This permits the AN to rationally and uniformly regulate multiple RISs to suppress interference among users in the service region and simultaneously boost the target signal of multiple users.
- We designed a DRL-based AN dynamic management scheme for multiple RISs. The scheme overcomes the technical challenge that exact channel state information cannot be obtained in real time in PMNs, which is required for traditional RIS management schemes.
- A numerical evaluation verifies the efficacy of the proposed RIS-assisted PMN downlink scheme in interference suppression. The results indicate that the communication capacity of the PMN can be substantially increased by deploying multiple RISs and controlling the RISs’ phase shifts and reflection coefficients.

The remaining sections of the paper are organized as follows: Section 2 summarizes the current status of knowledge. Section 3 analyzes the downlink transmission process within a PMN and formulates the multi-RIS management problem. This paper gives a succinct description of the A3C-based RIS management scheme in Section 4, which also serves as a brief introduction to the DRL. In Section 5, simulation results are presented and analyzed. Section 6 of this paper provides a summary of our work.

## 2. Related Works

In the recent literature, significant advancements have been made in various aspects of proactive mobile networks (PMNs), showcasing the growing interest and research efforts in this field. For instance, ref. [18] proposes expected mobility management, which answers the problem of network facilities in PMNs tracking the mobility of serviced SMs. Building upon this premise, ref. [8] presents a machine-centric proactive multi-cell association (PMCA) scheme that demonstrates the viability of an open-loop transmission-based architecture. With the aid of a proactive service and an edge server, a substantial study has been conducted on precaching relevant data near the user [9,19]. Regarding communication security, some studies have also proposed to achieve eavesdropping avoidance through proactive interference [20]. For the specific data transmission and resource management method, refs. [6,10,16] provide uplink and downlink solutions, respectively. The core challenge in the uplink is to ensure transmission reliability when the network is in passive service without control interaction. In addition to reliability, energy efficiency is also an important consideration in the downlink. Refs. [6,16] design a dual reinforcement learning iterative technique in a shared environment that realizes the reliability guarantee of uplink transmission in PMNs via free control interaction. Ref. [10] recommends that the SM controls the network side during downlink transmission, and by introducing non-real-time information in the preceding uplink process, it facilitates the selection of resources used in the present downlink transmission. Although research on PMNs is still in the exploratory stage, the proposed scheme has its limitations as it only examines performance from a single strategy.

In contrast, the technology of using RISs for auxiliary transmission has reached a relatively mature stage. Researchers have proposed innovative relay-assisted RIS structures, such as the one presented in [21], which connects parallel RISs via a full-duplex relay to reduce the number of reflective components required for the same rate. Refs. [22,23] examined the cooperation system composed of an RIS and decoding and forwarding relays in half-duplex and full-duplex operating modes, respectively. By combining an RIS and a relay into a cooperative system, the communication performance can be significantly enhanced. Additional gains can be obtained if the self-interference at the full-duplex relay is sufficiently suppressed. Refs. [24,25,26] investigated the communication performance of the cooperative system composed of multiple distributed RISs and relays. Refs. [27,28] proposed various cooperative system schemes. Compared to RIS-only or relay-only transmission schemes in various transmission environments, they all exhibited significant performance enhancements. Moreover, in schemes with relays, increasing the number of RIS components results in a greater gain than in schemes without relays. Ref. [29] proposed a novel RIS auxiliary communication system with the RIS controller functioning as a relay with decoding and forwarding capabilities. In contrast to the preceding cooperative system, the controller of the RIS is located within its near-field range. These advancements highlight the potential of RIS technology in enhancing the communication performance and promoting cooperative systems.

Furthermore, recent research has explored the application of RISs for interference mitigation, resulting in groundbreaking findings. Studies in [30] comprehensively examine the interference handling capacity of RIS-enhanced communication systems. The authors of [31] investigated an optimization problem involving phase shift design and beamforming strategies at all base stations in a multi-cell network powered by a single RIS as they delved deeper into the domain of inter-cell interference. Experts in [32] targeted systems assisted by RISs and interference, optimizing quasi-static phase shifts under both instantaneous and statistical channel state information (CSI) scenarios. Additionally, Ref. [33] implements RIS technology in high-speed railway networks to mitigate interference from intentional or unintentional sources, devising complex and sub-optimal algorithms to generate RIS phase shifts that maximize the signal-to-interference-plus-noise ratio.

These studies demonstrate that extensive research has been conducted on RIS technology and its potential to enhance communication performance and reduce interference. However, it is crucial to note that the studies mentioned above have predominantly focused on isolated instances of inter-user interference while disregarding the multi-user scenario. In addition, the conditions learned by their proposed schemes, which are founded on real-time CSI, need to be revised to meet the PMN requirements. Given the growing interest in and importance of research in the field of PMNs, there is an evident need for developing a novel scheme that can effectively address the PMNs’ particular requirements. In particular, this plan should consider the simultaneous scheduling of numerous RISs and address the challenges presented by the forbidding direct method for obtaining precise real-time CSI. This paper’s primary objective is to fill these gaps and provide a comprehensive solution for these critical PMN features.

## 3. System Model and Problem Formulation

**Figure 2:**
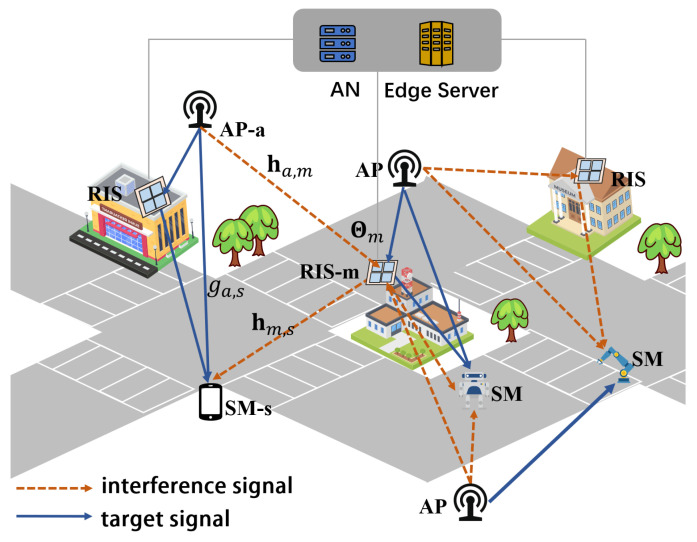
Introduction to multiple RIS-assisted PMN downlink processes. The AN controls and adjusts all RISs in a unified manner. The goal is to assist the multipath superposition enhancement of the target signal while allowing the interfering signals to superimpose and suppress each other.

This paper considers an RIS-assisted downlink in a proactive mobile network, as depicted in Figure 2, in which multiple RISs aid the transmission between *A* APs and *S* SMs. The region managed and served by a single AN has *M* RISs.

### 3.1. Channel Model

Suppose each AP and SM is equipped with only one single antenna, and each RIS consists of *N* reflecting elements. We denote the reflection coefficient matrix of the *m*-th RIS by $\Theta_{m}=\mathrm{diag}\left(\alpha_{m,1}e^{j\phi_{m,1}},\cdots ,\alpha_{m,N}e^{j\phi_{m,N}}\right)\in C^{N\times N}$. Here, $\alpha_{m,n}\in \left[0,1\right]$ and $\phi_{m,n}\in \left[0,2\pi \right)$ indicate the amplitude reflection coefficient and the phase shift of the *n*-th unit of the *m*-th RIS, separately. Let $h_{a,m}={\left[h_{a,m,1},\cdots ,h_{a,m,N}\right]}^{H}\in C^{N\times 1}$ and $h_{m,s}={\left[h_{m,s,1},\cdots ,h_{m,s,N}\right]}^{H}\in C^{N\times 1}$ denote the channel efficient of the AP-RIS link and the RIS-SM link, respectively. Furthermore, we use $g_{a,s}$ to denote the channel efficiency of the AP-SM direct link. $h_{a,m,n}$, $h_{m,s,n}$, and $g_{a,s}$ follow independent Rician fading as [30]

(1) $$h_{a,m,n}=\sqrt{\frac{\varpi_{a,m}}{\varpi_{a,m}+1}}{\bar{h}}_{a,m,n}+\sqrt{\frac{1}{\varpi_{a,m}+1}}{\hat{h}}_{a,m,n}$$

(2) $$h_{m,s,n}=\sqrt{\frac{\varpi_{m,s}}{\varpi_{m,s}+1}}{\bar{h}}_{m,s,n}+\sqrt{\frac{1}{\varpi_{m,s}+1}}{\hat{h}}_{m,s,n}$$

(3) $$g_{a,s}=\sqrt{\frac{\varpi_{a,s}}{\varpi_{a,s}+1}}{\bar{g}}_{a,s}+\sqrt{\frac{1}{\varpi_{a,s}+1}}{\hat{g}}_{a,s}$$

where $\varpi_{a,m}$, $\varpi_{m,s}$, and $\varpi_{a,s}$ are the corresponding Rician factors, respectively. ${\bar{h}}_{a,m,n}$, ${\bar{h}}_{m,s,n}$, and ${\bar{g}}_{a,s}$ are the line-of-sight (LoS) parts of the fading channel. Furthermore, ${\hat{h}}_{a,m,n}$, ${\hat{h}}_{m,s,n}$, and ${\hat{g}}_{a,s}$ are the non-line-of-sight (NLoS) parts.

For ${\bar{h}}_{a,m,n}$, we have

(4) $${\bar{h}}_{a,m,n}=\sqrt{\beta }d_{a,m}^{-\frac{\alpha_{0}}{2}}e^{-j\left(n-1\right)\pi \sin\theta_{a,m}}$$

where $\theta_{a,m}$ represents the angle of arrival (AoA) at the *m*-th RIS that the single sent by the *a*-th AP [34]. The $\alpha_{0}$ is the path loss exponent and the β denotes the path loss at the reference distance of 1 meter. $d_{a,m}$ is the distance between the RIS and the AP. ${\bar{h}}_{m,s,n}$ and ${\bar{g}}_{a,s}$ can be obtained similarly. However, $\theta_{m,s}$ and $\theta_{a,s}$ are the angle of departure (AoD). For the NLoS parts, we have ${\hat{h}}_{a,m,n}=d_{a,m}^{-\alpha /2}\dot{h}$, where $\dot{h}$ correspond to the complex Gaussian distribution CN(0,1). Both ${\hat{h}}_{m,s,n}$ and ${\hat{g}}_{a,s}$ are modelled similarly.

Since the mobility of the SMs, transmission delay, and processing delay cannot be neglected in the actual PMN, it is difficult to obtain the ideal CSI. If obsolete CSI is used to design the phase change, the performance loss will be glaring. Therefore, it is important to consider obsolete CSI in the RIS-assisted PMN system. In this scenario, τ represents the time difference between the obsolete CSI and the real-time CSI. The relation between the obsolete CSI $\tilde{h}\left[t-\tau \right]$ and the real-time CSI h[t] can then be expressed as [34]

(5) $$h\left[t\right]=\kappa \tilde{h}\left[t-\tau \right]+\sqrt{1-\kappa^{2}}\Delta \left(\tau \right),$$

where κ is the temporal correlation coefficient or the obsolete CSI coefficient, which is given by

(6) $$\kappa =J_{0}\left(2\pi f_{D}\tau \right),$$

where $J_{0}\left(\cdot \right)$ is the zeroth-order Bessel function of the first kind and $f_{D}$ is the Doppler shift. $f_{D}$ is calculated by $f_{D}=vf_{c}/c$, given the carrier frequency $f_{c}$, where *c* is the speed of light. In addition, Δ(τ) represents the error term, which is distributed independently from ${\tilde{h}}_{t-\tau }$ with zero-mean and $\sigma_{h}$ variance complex Gaussian entries.

### 3.2. RIS-Aided PMN Downlink Capacity

For the *s*-th SM, the received signal at time *t* can be written as

(7) $$y_{s}\left[t\right]=\sum_{a}\sqrt{p_{a}\left[t\right]}\left(\sum_{m}h_{m,s}^{H}\left[t\right]\Theta_{m}\left[t\right]h_{a,m}\left[t\right]+g_{a,s}\left[t\right]\right)x_{a}\left[t\right]+z\left[t\right],$$

where $x_{a}\left[t\right]$ denotes the desired signal sent by the *a*-th AP, z[t] corresponds to $CN(0,\sigma^{2})$, which denotes the noise, and $p_{a}\left[t\right]$ is the transmit power (in dBm) of the AP. Use $\Omega_{s}\left[t\right]$ to indicate the set of APs performing downlink transmission for the *s*-th SM. It follows that $\Omega_{i}\left[t\right]⋂\Omega_{j}\left[t\right]=⌀,1⩽i,j⩽S$ and ${⋃}_{i=1}^{S}\Omega_{i}\left[t\right]\subseteq \left\{1,\cdots ,A\right\}$. According to (7), we can therefore obtain the achievable rate of the RIS-aided PMN downlink transmission as

(8) $$R_{s}\left[t\right]=\log\left(1+\frac{\sum_{a\in \Omega_{s}\left[t\right]}p_{a}\left[t\right]\left(\sum_{m}h_{m,s}^{H}\left[t\right]\Theta_{m}\left[t\right]h_{a,m}\left[t\right]+g_{a,s}\left[t\right]\right)Q_{a}\left[t\right]\left(g_{a,s}\left[t\right]+\sum_{m}h_{m,s}\left[t\right]\Theta_{m}^{H}\left[t\right]h_{a,m}^{H}\left[t\right]\right)}{\sum_{b\notin \Omega_{s}\left[t\right]}p_{a}\left[t\right]\left(\sum_{m}h_{m,s}^{H}\left[t\right]\Theta_{m}\left[t\right]h_{b,m}\left[t\right]+g_{b,s}\left[t\right]\right)Q_{b}\left[t\right]\left(g_{b,s}\left[t\right]+\sum_{m}h_{m,s}\left[t\right]\Theta_{m}^{H}\left[t\right]h_{b,m}^{H}\left[t\right]\right)+\sigma^{2}}\right).$$

In (8), $Q_{a}\left[t\right]=E\left[x_{a}\left[t\right]{\bar{x}}_{a}\left[t\right]\right]$. When the phase shifts are fixed in the maximal interference situation, $Q_{a}^{*}\left[t\right]$ is expressed by

(9) $$Q_{a}^{*}\left[t\right]=\frac{\left(\sum_{m}h_{m,s}^{H}\left[t\right]\Theta_{m}\left[t\right]h_{a,m}\left[t\right]+g_{a,s}\left[t\right]\right)\left(g_{a,s}\left[t\right]+\sum_{m}h_{m,s}\left[t\right]\Theta_{m}^{H}\left[t\right]h_{a,m}^{H}\left[t\right]\right)}{∥\sum_{m}h_{m,s}^{H}\left[t\right]\Theta_{m}\left[t\right]h_{a,m}\left[t\right]+g_{a,s}{\left[t\right]∥}^{2}}.$$

Obviously, $Q_{a}\left[t\right]⩽Q_{a}^{*}\left[t\right]⩽1$, and the same applies to $Q_{b}\left[t\right]$. Thus, the RIS-aided PMN downlink capacity for the *s*-th SM is given by

(10) $$C_{s}\left[t\right]=\log\left(1+\frac{\sum_{a\in \Omega_{s}\left[t\right]}p_{a}\left[t\right]{∥\sum_{m}h_{m,s}^{H}\left[t\right]\Theta_{m}\left[t\right]h_{a,m}\left[t\right]+g_{a,s}\left[t\right]∥}^{2}}{\sum_{b\notin \Omega_{s}\left[t\right]}p_{b}\left[t\right]{∥\sum_{m}h_{m,s}^{H}\left[t\right]\Theta_{m}\left[t\right]h_{b,m}\left[t\right]+g_{b,s}\left[t\right]∥}^{2}+\sigma^{2}}\right).$$

It should be pointed out that the RIS-aided PMN downlink process can only acquire the estimated CSI $\tilde{h}\left[t\right]$. Thus, the capacity in (10) is calculated based on the actual CSI expressed by (5).

### 3.3. Optimization Problem Formulation

According to (10), the reflection coefficient matrices of the RISs play a crucial role in the interference capacity of RIS-aided PMN networks. To enhance the desired signal and reduce interference, it is necessary to design optimal RIS phase shifts and amplitude reflection coefficients. In order to achieve capacity assisted by an RIS, the following capacity maximization problem is formulated:

(11) $$\begin{array}{cc} P: & \;\;\max_{\left\{\alpha_{m,n}\right\},\left\{\phi_{m,n}\right\}}\;\min_{s}\;\frac{1}{T}\sum_{t=1}^{T}C_{s}\left[t\right]\\ s.t. & \begin{array}{c} c1:0⩽\alpha_{m,n}\left[t\right]⩽1,\\ c2:0⩽\phi_{m,n}\left[t\right]<2\pi . \end{array} \end{array}$$

In (11), we take into account the fairness of the network used by each SM and establish the objective as maximizing the minimal capacity of all devices. We further note that the log function increases monotonically. The above optimization is equivalently transformed to

(12) $$\begin{array}{cc} P: & \;\;\max_{\left\{\alpha_{m,n}\right\},\left\{\phi_{m,n}\right\}}\;\min_{s}\;\frac{1}{T}\sum_{t=1}^{T}\left(\frac{\sum_{a\in \Omega_{s}\left[t\right]}p_{a}\left[t\right]{∥\sum_{m}h_{m,s}^{H}\left[t\right]\Theta_{m}\left[t\right]h_{a,m}\left[t\right]+g_{a,s}\left[t\right]∥}^{2}}{\sum_{b\notin \Omega_{s}\left[t\right]}p_{b}\left[t\right]{∥\sum_{m}h_{m,s}^{H}\left[t\right]\Theta_{m}\left[t\right]h_{b,m}\left[t\right]+g_{b,s}\left[t\right]∥}^{2}+\sigma^{2}}\right)\\ s.t. & \begin{array}{c} c1:0⩽\alpha_{m,n}\left[t\right]⩽1,\;\;c2:0⩽\phi_{m,n}\left[t\right]<2\pi . \end{array} \end{array}$$

We can see that problem (12) is a fractional optimization problem, a difficult-to-solve non-convex problem. In addition, given the practical significance of the problem, it is costly for the AN to traverse all network states in real time for each time slot in a highly dynamic environment to calculate and locate the optimal point. In order to overcome this difficulty, we propose an algorithmic computation taking advantage of DRL.

## 4. Deep Reinforcement Learning Approach

This section begins by demonstrating how to formulate problem (12) as a reinforcement learning problem. Furthermore, based on the characteristics of the problem’s continuous high-dimensional decision variables, a scheme based on A3C is proposed to modify the RISs phase shifts and amplitude reflection coefficient.

### 4.1. Reinforcement Learning Problem Formulation

Various factors, such as the fluctuating network load, the state of wireless channels, and the transmission requirements of multiple devices, exhibit statistical patterns and state transition characteristics over time in practical network scenarios. From an engineering standpoint, the significance of (12) resides in its ability to guide decision making regarding the network-dependent behaviors of RISs. Given these conditions’ inherent uncertainty and stochastic nature, numerous decision-making problems can be effectively addressed by transforming them into Markov decision process (MDP) problems and applying RL theory to maximize decision-making utility. Unlike conventional stochastic optimal control methods [35], RL approaches offer distinct benefits by eliminating the need for extensive prior knowledge of system dynamics or objectives [36,37,38]. Instead, RL strategies discover optimal control policies via direct interaction with the system. Consequently, the first step is to re-formulate (12) as an MDP problem.

The MDP is expressed by a five-tuple 〈S,A,P,R,ε〉, where S is the set of observed environment states, A is a set of available actions for the agent, P denotes state transition probabilities, R is the reward function, and ε∈[0,1] indicates the discount factor. For each step, the agent takes an action $a_{t}\in A$ according to the environment states $s_{t}\in S$. The action affects the state’s transition to a new $s_{t+1}$ while giving the agent a certain reward $r_{t}=R\left(s_{t},a_{t}\right)$. The MDP components will be described in the following.

**State Space:** At the beginning of time *t*, AN obtains the spatial position $s_{t}^{p}$ of all SMs, all required channel information $s_{t}^{c}$, and service relationship between APs and SMs during downlink transmission $s_{t}^{s}$ in the region by means of the sensors and historical data under its jurisdiction. $s_{t}^{p}$ is denoted as

(13) $$s_{t}^{p}=\left\{p_{x}^{1}\left[t\right],\cdots .,p_{x}^{S}\left[t\right],p_{y}^{1}\left[t\right],\cdots ,p_{y}^{S}\left[t\right]\right\},$$

where $p_{x}^{s}\left[t\right]$ and $p_{y}^{s}\left[t\right]$, respectively, represent the horizontal and vertical coordinates of the *s*-th SM. $s_{t}^{c}$ contains the channel information of all AP-RIS links, AP-SM links, and RIS-SM links, which is given by

(14) $$s_{t}^{c}=\left\{h_{a,m},h_{m,s},g_{a,s}\right\}.$$

$s_{t}^{s}$ is represented by a vector, with each element corresponding to the SM served by the *s*-th AP during the slot. To this end, the state space at time t is defined as

(15) $$s_{t}=s_{t}^{p}⋃s_{t}^{c}⋃s_{t}^{s}.$$

Two more issues should be noted. Adding irrelevant or weakly correlated features to the state will undoubtedly increase efforts of data collection and likely decrease the system’s performance. As a result, we amend the consideration of SMs mobility norms and service relationship modifications in subsequent simulations [39,40]. However, because this work is not centered on this subject, it will not be discussed in detail here. The second issue is that, in this work, the imaginary portion of the channel coefficients will be converted to real integers. Then, these coefficients and the rear portion of the channel coefficients can be fed into the neural network [41].

**Action Space:** According to the present state of the RIS-assisted PMN downlink system, decisions must be made regarding the phase shift and amplitude reflection coefficient. Consequently, the action space is represented by

(16) $$a_{t}=\left[\alpha_{1,1},\cdots ,\alpha_{1,N},\alpha_{2,1},\cdots ,\alpha_{M,N},\phi_{1,1},\cdots ,\phi_{1,N},\phi_{2,1},\cdots ,\phi_{M,N}\right].$$

**State Transition Probability:** In the absence of prior knowledge of the probability of state transitions, the agent determines $P(s_{t+1}|\left(s_{t},a_{t}\right))$ based solely on the environment [42]. $P(s_{t+1}|\left(s_{t},a_{t}\right))$ represents the probability distribution of $s_{t+1}$ for the given $s_{t}$ and the chosen $a_{t}$. In this study, the transition on the channels, such as (1)–(5), and the spatial location of SMs and the transfer of service correspondence relationship with APs depend on the simulation setting.

**Reward Function:** The reward function, which represents the immediate reward for a given state action dyad, is generally related to the objective function. This paper aims to maximize the minimal capacity of all SMs within RIS-assisted PMN downlink transmissions with mutual interference. Therefore, the reward function is determined by

(17) $$r_{t}=\min C_{s}\left[t\right]$$

Using the above entry, π denotes the strategy of the AN choosing action by the network status. Thus, the total expected reward for the future by one action is the Q-function,

(18) $$Q_{\pi }\left(s^{\prime },a^{\prime }\right)=E_{\pi }\left[R_{t}|s_{0}=s^{\prime },a_{0}=a^{\prime }\right]=E_{\pi }\left[\sum_{t=0}^{\infty }\varepsilon^{t}\cdot r\left(s_{t},a_{t}\right)|s_{0}=s^{\prime },a_{0}=a^{\prime }\right],$$

where $R_{t}$ is the discounted accumulated reward, indicating how the future rewards influence the current state value. Sometimes, the expectation of a certain state’s future reward is directly measured, that is,

(19) $$V_{\pi }\left(s^{\prime }\right)=E_{\pi }\left[Q_{\pi }\left(s^{\prime },a^{\prime }\right)|s_{0}=s^{\prime }\right]=\sum_{a}\pi \left(a|s^{\prime }\right)Q_{\pi }\left(s^{\prime },a\right)$$

Then, the RL aims to find the optimal strategy $\pi^{*}$ that for every $s^{\prime }$ and $a^{\prime }$

(20) $$\pi^{*}=\arg\max_{\pi }Q_{\pi }\left(s^{\prime },a^{\prime }\right)=\arg\max_{\pi }V_{\pi }\left(s^{\prime }\right)$$

### 4.2. Actor–Critic Decision Framework

Due to the high dimensionality and continuity of the state and action spaces in this problem, the above *Q*-function and optimal strategy are challenging to solve directly. This suggests approximating the relationship between S, A, and *Q*-functions using parameterized functions. Deep neural networks (DNNs) have excellent fitting functions [43,44]. In contrast to supervised learning and other training methods with distinct objectives, however, there is no obvious objective when a DNN is used to depict the relationship between elements in RL. The actor–critic structure is therefore employed [45].

The high dimensionality and continuity of the state and action spaces pose significant challenges in directly solving the *Q*-function and optimal strategy in this problem. To address this, an alternative approach is to approximate the relationship between the state space S, action space A, and *Q*-functions using parameterized functions. A deep neural network (DNN) is well suited for this task due to its excellent function approximation capabilities. However, unlike supervised learning or other training methods with explicit objectives, there is no clear objective when using a DNN to represent the relationship between elements in reinforcement learning (RL). To overcome this, an actor–critic structure is employed, as suggested in [45]. The actor–critic architecture combines both policy evaluation (the critic) and policy improvement (the actor) to learn and optimize the policy in an RL setting. This allows for effective training of the DNN and facilitates the approximation of the complex relationships between states, actions, and *Q*-values, leading to an improved performance in solving the problem at hand.

The agent in the AC framework consists of an actor and a critic. The actor is a DNN that corresponds to a strategy function whose purpose is to solve the problem of continuous action selection by utilizing the parametric properties of DNNs and probability actions. The parameters in this section are represented by θ, and the approximate strategy function can be written as follows:

(21) π(s,θ)=P[a|s,θ]≈π(a|s).

The critic is an additional DNN that utilizes the *Q*-function. It is capable of solving expected return evaluations on high-dimensional continuous state spaces. Specifically, $\hat{Q}\left(s,a,w\right)\approx Q_{\pi }\left(s,a\right)$, where w is the parameter for this part. The actor in the actor–critic framework executes an action based on the current strategy in response to the current state during each training episode. The environment then changes state and rewards the critic with feedback. Using the temporal difference (TD) algorithm, the critic, responsible for evaluating the quality of the actor’s actions, is updated to improve its judgment and evaluation capabilities. The actor is modified using the policy gradient method to optimize for higher returns. However, it is important to note that in the base version of the actor–critic architecture, both the actor and the critic rely on gradient updates and are interdependent, making convergence to a stable solution challenging. The interaction between these two components can result in instability and training difficulties for DNNs.

The asynchronous advantage actor–critic (A3C) algorithm builds upon the actor–critic algorithm by introducing concurrent actors and asynchronous training of neural networks. This key distinction significantly accelerates the convergence process [46]. In the A3C algorithm, the network parameters are stored on a central server. Each actor operates independently and interacts with the environment, collecting experiences and generating gradients based on their local network. Once an actor reaches a terminal state or the maximum action index, it transmits its gradients to the central server. The central server then updates the global parameters using these gradients and redistributes the updated parameters to all the actors. This ensures that all actors share the same policy while avoiding high parameter correlation that can arise with a single agent. Unlike traditional deep Q-networks (DQNs), A3C does not require a replay memory [46]. Additionally, the training duration can be drastically reduced.

### 4.3. A3C-Based Approach

In the following is a description of the implementation of the A3C-based orchestration solution illustrated in Algorithm 1. When the environment is in state $s_{t}$, the estimated state value is $V_{\pi }\left(s_{t};\omega \right)$ in each time slot *t*, and the agent executes action $a_{t}$ according to policy $\pi (a_{t}|s_{t};\theta )$. When the utmost number of steps is reached, or the final state is attained, the policy and its corresponding value function are iterated and updated. Then, A3C uses a k-step reward for parameter updating, which is provided by

(22) $$R_{t}=\sum_{i=0}^{k-1}\varepsilon^{i}\cdot r_{t+i}+\varepsilon^{k}\cdot V_{\pi }\left(s_{t+k};\omega \right)$$

where *k* is the variation from state to state, $t_{max}$ is the upper limit, and ε is the discount factor signifying how future rewards affect the current state value [46,47].
**Algorithm 1:** A3C-Based Solution
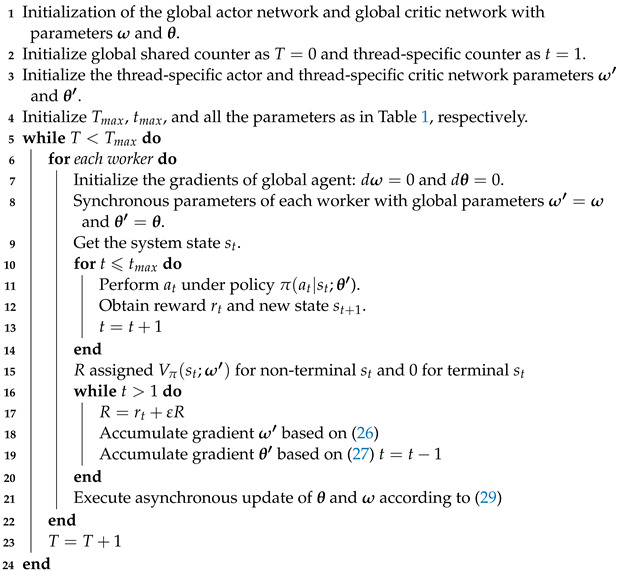


Similar to the AC algorithm, A3C specifies the advantage function $A_{t}$ to reduce the estimation variance, which is given by

(23) $$A\left(s_{t},a_{t};\theta ,\omega \right)=R_{t}-V_{\pi }\left(s_{t};\omega \right)$$

where θ and ω are actor and critic network parameters, respectively. Therefore, the advantage function $A_{t}$ can be used to enhance the learning capacity of agents to prevent them from over- or under-estimating the action. In addition, based on the advantage function $A_{t}$, the loss function of the actor network can be given by

(24) $$L_{\pi }\left(\theta \right)=\log\pi \left(a_{t}|s_{t};\theta \right)A\left(s_{t},a_{t};\theta ,\omega \right)+\zeta H\left(\pi \left(s_{t};\theta \right)\right),$$

The term $H(\pi \left(s_{t};\theta \right))$ is incorporated in the training process to promote exploration and prevent premature convergence. Additionally, the parameter ζ is utilized to regulate the strength of entropy regularization, which helps balance the exploration/exploitation tradeoff. The critic network’s approximated value loss function is represented as:

(25) $$L_{V}\left(\omega \right)={\left(R_{t}-V_{\pi }\left(s_{t};\omega \right)\right)}^{2},$$

which is used to update the value function $V_{\pi }\left(s_{t};\omega \right)$. The critic network undergoes updates through the use of a cumulative gradient which is as follows:

(26) $$d\omega \leftarrow d\omega +\frac{\partial R_{t}-V_{\pi }\left(s_{t};\omega \right)}{\partial \omega^{\prime }}$$

Next, the actor network is updated and iterated through

(27) $$d\theta \leftarrow d\theta +\nabla_{\theta^{\prime }}\log\pi \left(a_{t}|s_{t};\theta^{\prime }\right)A\left(s_{t},a_{t};\theta^{\prime },\omega^{\prime }\right)+\zeta \nabla_{\theta^{\prime }}H\left(\pi \left(s_{t};\theta^{\prime }\right)\right).$$

Furthermore, the parameters $\theta^{\prime }$ and $\omega^{\prime }$ relate to specific workers, whereas the parameters θ and ω correspond to the global actor and critic network, respectively.

**Figure 3:**
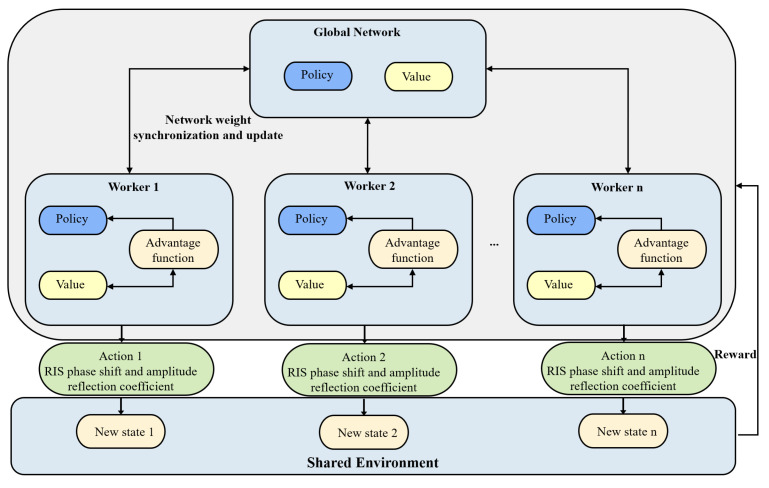
A3C-based framework for management phase shifts and amplitude reflection coefficients of the RISs at the ANs.

In our training process, we rely on the traditional non-centered RMSProp algorithm [48]. This includes reducing the two loss functions and adjusting the actor and critic parameters using their accumulated gradients, as depicted in Equations (26) and (27). The gradient computed via RMSProp can be represented as follows:

(28) $$q\leftarrow \xi q+\left(1-\xi \right)d{\left(*\right)}^{2}$$

where ξ is the momentum and d(∗) is the accumulated gradients of the policy or value loss function. Based on the obtained *q*, the update is performed according to

(29) $$*\leftarrow *-\sigma \frac{d(*)}{\sqrt{q+\epsilon }}$$

where σ is the learning rate and ϵ is a tiny positive number used to avoid errors when the denominator equals 0 [44]. The global framework of the A3C algorithm in this paper is illustrated in Figure 3.

## 5. Analysis of Simulation Results

### 5.1. Simulation Setting

All simulations were conducted inside a 200m×200m rectangular region where all wireless networks are believed to be controlled by one AN. There are 30 APs randomly deployed in the region. The transmit power of each AP is set to not exceed 32 dBm. During the downlink process, the AN randomly selects one of the three APs closest to each SM to serve the transmission of the SM. In addition, due to the mobility of the SM, if the closest three APs around it all have been selected to serve other SMs, the AP closest to it is used and is not assigned to perform this time downlink. The path loss factor $\alpha_{0}$ is 2 and β=−25 dB. In addition, the noise power density is $\sigma^{2}=-110$ dBm. The moving speed of all SMs in the area is limited to 40 km/h–100 km/h, and one of the eight directions can be randomly selected to move every minute. The default values of other parameters are summarized in Table 1.

**Table 1:** Important parameters in the simulation setup.

Parameter	Value
The Rician factors $\varpi_{a,m}$, $\varpi_{m,s}$, and $\varpi_{a,s}$	(4, 5, 6)
The temporal correlation coefficient κ	0.7
Number of APs *A*	20
Number of SMs *S*	18
Number of elements in each RIS *N*	32
Discount factor ε	0.8
Coefficient ξ	0.1, 0.001, 0.0001
Noise power density $\sigma^{2}$	−164 dBm/Hz
Max transmit power of each AP	27 dBm

Furthermore, in the A3C scheme, we configure the hidden layer of the DNN to be a fully connected layer whose active function is relu. For the actor, the number of hidden layers is set to 3, with 300, 400, and 200 neurons in each hidden layer, respectively. The number of neurons in each of the critic’s four hidden layers is 400, 500, 500, and 300, respectively.

It should be noted that our simulation only approximates the DNN network structure and parameters based on the existing literature, particularly [49,50,51]. Our aim is to assess the efficacy of our proposed scheme. Nonetheless, to achieve more favorable outcomes, it is imperative to conduct further research to optimize other DNN hyperparameters in RL and explore alternative network structures such as LSTM and RNNs. This paper does not delve into this aspect of the topic.

### 5.2. Results and Analysis

**Figure 4:**
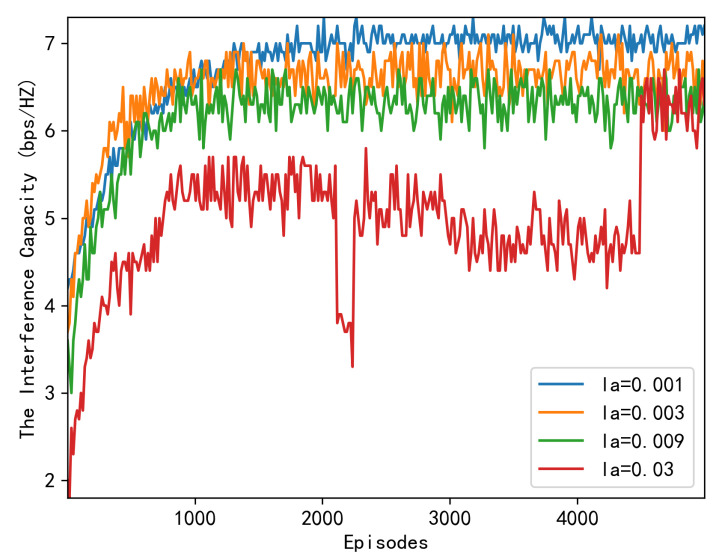
Downlink capacity over episodes under different learning rates $l_{a}$.

**Figure 5:**
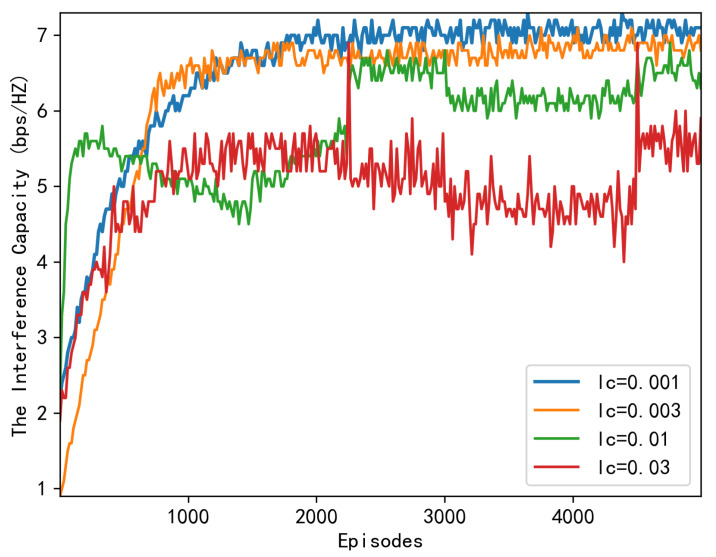
Downlink capacity over episodes under different learning rates $l_{c}$.

We begin by demonstrating the convergence of our proposed algorithm at various learning rates. Figure 4 depicts convergence under varying actor learning rates $l_{a}$, with the critic’s learning rate set to $l_{c}=0.001$, whereas Figure 5 depicts convergence under varying critic learning rated, with the actor’s learning rate set to $l_{a}=0.001$. As can be seen in these two figures, the system reward initially increases abruptly. Then, it converges at nearly 3000 episodes under various learning rate combinations, indicating that our proposed algorithm converges rapidly. Specifically, when the learning rate is 0.03, although it achieves a swift convergence, its capacity performance is inferior to that of the 0.001 case. It is no surprise that an appropriate learning rate should be selected for convergence speed.

We will now assess the effectiveness of our proposed scheme, which is based on A3C. To compare its performance, we will consider three baseline methods:

1. **Without RIS**: This scenario depicts the PMN downlink transmission in its original state, without any interference suppression mechanisms [10]. In this case, RIS-related processes are eliminated and the interference capacity is provided by

(30) $$C_{s}\left[t\right]=\log\left(1+\frac{\sum_{a\in \Omega_{s}\left[t\right]}p_{a}\left[t\right]{∥g_{a,s}\left[t\right]∥}^{2}}{\sum_{b\notin \Omega_{s}\left[t\right]}p_{b}\left[t\right]{∥g_{b,s}\left[t\right]∥}^{2}+\sigma^{2}}\right).$$

2. **Unify reflecting coefficients and random phase shift**: In this case, we consider introducing an RIS to be deployed in the region to assist the downlink process. However, there is no effective management mechanism, and the components in the RIS can only be randomly configured [49]. In this method, the amplitude reflection coefficient of all RISs is set to 1, and the phase shifts of RISs are designed randomly according to a uniform distribution in [0,2π).

3. **Maximizing Receiving Power**: This method seeks to maximize the received power of the target signal at the SM by devising the RIS phase shift and reflection factor while disregarding mutual interference between SMs, that is

(31) $$\begin{array}{cc} P: & \;\;\max_{\left\{\alpha_{m,n}\right\},\left\{\phi_{m,n}\right\}}\;\min_{s}\;\frac{1}{T}\sum_{t=1}^{T}\sum_{a\in \Omega_{s}\left[t\right]}p_{a}\left[t\right]{∥\sum_{m}h_{m,s}^{H}\left[t\right]\Theta_{m}\left[t\right]h_{a,m}\left[t\right]+g_{a,s}\left[t\right]∥}^{2}\\ s.t. & \begin{array}{c} c1:0⩽\alpha_{m,n}\left[t\right]⩽1,c2:0⩽\phi_{m,n}\left[t\right]<2\pi . \end{array} \end{array}$$

This problem has been solved with the method in [52].

**Figure 6:**
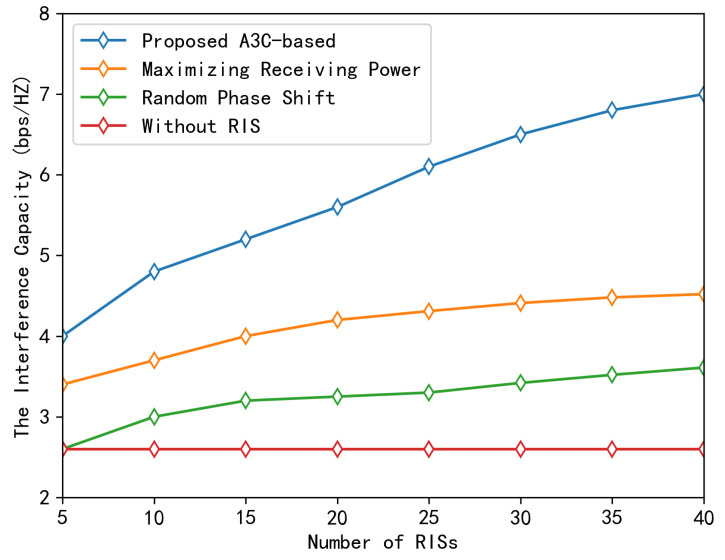
Downlink capacity under mutual interference among SMs against the number of RISs.

Figure 6 depicts the effect of deploying various RISs in the experimental region on the downlink transmission capacity convergence performance. The results indicate that increasing the number of RISs deployed enables the system performance to converge towards higher capacities. Nonetheless, the capacity advantage diminishes as the number of RISs increases. In addition, the proposed A3C-based scheme has clear performance advantages over the other three baseline schemes, with a 173% improvement in performance over the scheme without RISs. Compared to the strategy of merely increasing the signal’s intensity without interference suppression, the performance is increased by 64%.

It is clear that the implementation of multiple RISs will improve the downlink transmission performance of the PMN system. However, the system’s channel capacity will be limited without an effective management mechanism, resulting in a random phase shift. A simple scheduling method, which aims to maximize the power of the target signal, can increase the system’s upper capacity limit. However, this may cause interference with other users, resulting in the performance curve stabilizing prematurely after reaching a certain value. To address this, the proposed A3C-based scheme is highly effective as it minimizes user interference and improves the quality of the received signal intended for the target.

**Figure 7:**
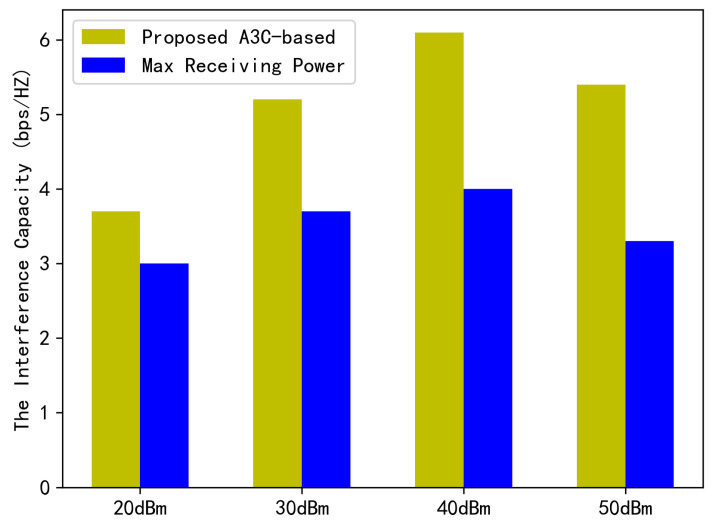
Downlink capacity under mutual interference among SMs against the transmission power.

We conducted an extensive analysis to determine how the strength of the target signal and interference signal affect the system capacity. We specifically compared the performance of the “Maximizing Receiving Power” case with our proposed “A3C-based” solution, which takes interference suppression into account. The results are presented in Figure 7. Our findings show that the system capacity changes as the AP transmit power increases. Generally, the capacity increases with power, but if it becomes too high, the capacity starts to decrease. This highlights the importance of optimizing the transmit power to balance capacity and avoid negative effects. Additionally, our results demonstrate that the proposed A3C-based scheme significantly improves the system performance compared to the baseline scheme. It achieves an impressive 71% improvement in system performance, emphasizing the importance of considering interference suppression when designing a downlink transmission scheme that caters to the PMN’s unique characteristics.

It is important to note that we have only verified the proposed plan’s effectiveness. However, there are various crucial factors to consider when it comes to actual deployment. These include the duration from policy training to stability convergence, limitations in equipment computing power, optimizing the targeted DNN structure, and ensuring that the samples are complex enough during online training via interaction with the environment. These issues require further attention and investigation.

## 6. Conclusions

In this paper, we present a solution to eliminate interference in the proactive mobile network downlink process. Our proposed method effectively reduces interference and improves the reliable capacity of the system by introducing RIS-aided technology. We formulate an optimization problem to design the phase shifts and reflection coefficients at multiple RISs. By using deep reinforcement learning as an A3C-based method, we solved the optimization problem in a time-varying and complex PMN environment where real-time channel state information is not readily available. The simulation results show that deploying RISs significantly enhances interference suppression, and our proposed scheme obtains greater capacity than baseline schemes. As the number of RISs increases, the capacity approaches its maximum, demonstrating the scalability and efficacy of our solution. These results indicate that RISs and DRL techniques can be incorporated into PMNs to facilitate exceptionally low-latency communication and improve the overall network performance. However, some details still need to be further studied, such as optimizing the DNN structure in the scheme and considering computing power factors in specific practical networks. Our follow-up work will continue to explore these areas in more depth.

## Data Availability

Not applicable.

## Abbreviations

The following abbreviations are used in this manuscript:

URLLC	Ultra-Reliable Low-Latency Communication
PMN	Proactive Mobile Network
DRL	Deep Reinforcement Learning
A3C	Asynchronous Advantage Actor–Critic
RIS	Reconfigurable Intelligent Surfaces
RAN	Radio Access Network
AP	Access Point
AN	Anchor Node
SM	Smart Machine
PMCA	Proactive Multi-Cell Association
CSI	Channel State Information
MDP	Markov Decision Process
DNN	Deep Neural Network

## References

[1] Park J., Samarakoon S., Shiri H., Abdel-Aziz M.K., Nishio T., Elgabli A., Bennis M. (2020). Extreme URLLC: Vision, challenges, and key enablers. arXiv.

[2] Eum S., Arakawa S., Murata M. A probabilistic Grant Free scheduling model to allocate resources for eXtreme URLLC applications. Proceedings of the 2022 IEEE Latin-American Conference on Communications (LATINCOM).

[3] Shi H., Zheng W., Liu Z., Ma R., Guan H. (2023). Automatic Pipeline Parallelism: A Parallel Inference Framework for Deep Learning Applications in 6G Mobile Communication Systems. IEEE J. Sel. Areas Commun..

[4] 3GPP (2019). Study on enhancement of Ultra-Reliable Low-Latency Communication (URLLC) Support in the 5G Core Network (5GC). Technical Report (TR) 23.725, 3rd Generation Partnership Project (3GPP), Version 16.2.0..

[5] Chen K.C., Zhang T., Gitlin R.D., Fettweis G. (2018). Ultra-low latency mobile networking. IEEE Netw..

[6] Wang Y., Chen K.C., Gong Z., Cui Q., Tao X., Zhang P. (2022). Reliability-Guaranteed Uplink Resource Management in Proactive Mobile Network for Minimal Latency Communications. IEEE Trans. Wirel. Commun..

[7] Cui Q., Zhang J., Zhang X., Chen K.C., Tao X., Zhang P. (2020). Online anticipatory proactive network association in mobile edge computing for IoT. IEEE Trans. Wirel. Commun..

[8] Liu C.H., Liang D.C., Chen K.C., Gau R.H. (2021). Ultra-Reliable and Low-Latency Communications Using Proactive Multi-Cell Association. IEEE Trans. Commun..

[9] Alqahtani F., Al-Maitah M., Elshakankiry O. (2022). A proactive caching and offloading technique using machine learning for mobile edge computing users. Comput. Commun..

[10] Wang X., Wang Y., Cui Q., Chen K.C., Ni W. (2022). Machine Learning Enables Radio Resource Allocation in the Downlink of Ultra-Low Latency Vehicular Networks. IEEE Access.

[11] Louie R.H., McKay M.R., Collings I.B. (2010). Open-loop spatial multiplexing and diversity communications in ad hoc networks. IEEE Trans. Inf. Theory.

[12] Zheng C., Zheng F.C., Luo J., Feng D. (2021). Open-loop communications for up-link URLLC under clustered user distribution. IEEE Trans. Veh. Technol..

[13] Hunter A.M., Andrews J.G., Weber S. (2008). Transmission capacity of ad hoc networks with spatial diversity. IEEE Trans. Wirel. Commun..

[14] Vaze R., Heath R.W. (2012). Transmission capacity of ad hoc networks with multiple antennas using transmit stream adaptation and interference cancellation. IEEE Trans. Inf. Theory.

[15] Cui Q., Gong Z., Ni W., Hou Y., Chen X., Tao X., Zhang P. (2019). Stochastic online learning for mobile edge computing: Learning from changes. IEEE Commun. Mag..

[16] Wang Y., Cui Q., Chen K.C. Machine Learning Enables Predictive Resource Recommendation for Minimal Latency Mobile Networking. Proceedings of the 2021 IEEE 32nd Annual International Symposium on Personal, Indoor and Mobile Radio Communications (PIMRC).

[17] Liu X., Liu Y., Chen Y. (2020). Machine learning empowered trajectory and passive beamforming design in UAV-RIS wireless networks. IEEE J. Sel. Areas Commun..

[18] Lin C.Y., Chen K.C., Wickramasuriya D., Lien S.Y., Gitlin R.D. Anticipatory Mobility Management by Big Data Analytics for Ultra-Low Latency Mobile Networking. Proceedings of the 2018 IEEE International Conference on Communications (ICC).

[19] Musa S.S., Zennaro M., Libsie M., Pietrosemoli E. (2022). Mobility-aware proactive edge caching optimization scheme in information-centric iov networks. Sensors.

[20] Zhang M., Yi H., Chen Y., Tao X. Proactive eavesdropping via jamming for power-limited UAV communications. Proceedings of the 2019 IEEE International Conference on Communications Workshops (ICC Workshops).

[21] Ying X., Demirhan U., Alkhateeb A. (2020). Relay aided intelligent reconfigurable surfaces: Achieving the potential without so many antennas. arXiv.

[22] Abdullah Z., Chen G., Lambotharan S., Chambers J.A. (2020). A hybrid relay and intelligent reflecting surface network and its ergodic performance analysis. IEEE Wirel. Commun. Lett..

[23] Abdullah Z., Chen G., Lambotharan S., Chambers J.A. (2020). Optimization of intelligent reflecting surface assisted full-duplex relay networks. IEEE Wirel. Commun. Lett..

[24] Yang L., Yang Y., da Costa D.B., Trigui I. (2020). Outage probability and capacity scaling law of multiple RIS-aided networks. IEEE Wirel. Commun. Lett..

[25] Do T.N., Kaddoum G., Nguyen T.L., Da Costa D.B., Haas Z.J. (2021). Multi-RIS-aided wireless systems: Statistical characterization and performance analysis. IEEE Trans. Commun..

[26] Zhang Y., Zhang J., Di Renzo M., Xiao H., Ai B. (2022). Reconfigurable intelligent surfaces with outdated channel state information: Centralized vs. distributed deployments. IEEE Trans. Commun..

[27] Huang C., Chen G., Gong Y., Wen M., Chambers J.A. (2021). Deep reinforcement learning-based relay selection in intelligent reflecting surface assisted cooperative networks. IEEE Wirel. Commun. Lett..

[28] Elhattab M., Arfaoui M.A., Assi C., Ghrayeb A. (2021). Reconfigurable intelligent surface enabled full-duplex/half-duplex cooperative non-orthogonal multiple access. IEEE Trans. Wirel. Commun..

[29] Zheng B., Zhang R. (2021). IRS meets relaying: Joint resource allocation and passive beamforming optimization. IEEE Wirel. Commun. Lett..

[30] Du L., Shao S., Yang G., Ma J., Liang Q., Tang Y. (2021). Capacity characterization for reconfigurable intelligent surfaces assisted multiple-antenna multicast. IEEE Trans. Wirel. Commun..

[31] Pan C., Ren H., Wang K., Xu W., Elkashlan M., Nallanathan A., Hanzo L. (2020). Multicell MIMO communications relying on intelligent reflecting surfaces. IEEE Trans. Wirel. Commun..

[32] Jia Y., Ye C., Cui Y. (2020). Analysis and optimization of an intelligent reflecting surface-assisted system with interference. IEEE Trans. Wirel. Commun..

[33] Ma Z., Wu Y., Xiao M., Liu G., Zhang Z. (2021). Interference suppression for railway wireless communication systems: A reconfigurable intelligent surface approach. IEEE Trans. Veh. Technol..

[34] Xia X., Xu K., Zhao S., Wang Y. (2020). Learning the time-varying massive MIMO channels: Robust estimation and data-aided prediction. IEEE Trans. Veh. Technol..

[35] Fleming W.H., Rishel R.W. (2012). Deterministic and Stochastic Optimal Control.

[36] Cui Q., Zhao X., Ni W., Hu Z., Tao X., Zhang P. (2022). Multi-Agent Deep Reinforcement Learning-Based Interdependent Computing for Mobile Edge Computing-Assisted Robot Teams. IEEE Trans. Veh. Technol..

[37] Zhang D., Zheng Z., Jia R., Li M. Visual tracking via hierarchical deep reinforcement learning. Proceedings of the Thirty-Fifth AAAI Conference on Artificial Intelligence.

[38] El-Bouri R., Eyre D., Watkinson P., Zhu T., Clifton D. Student-teacher curriculum learning via reinforcement learning: Predicting hospital inpatient admission location. Proceedings of the 37th International Conference on Machine Learning (PMLR).

[39] Meng F., Chen P., Wu L., Cheng J. (2020). Power allocation in multi-user cellular networks: Deep reinforcement learning approaches. IEEE Trans. Wirel. Commun..

[40] Cui Q., Hu X., Ni W., Tao X., Zhang P., Chen T., Chen K.C., Haenggi M. (2022). Vehicular mobility patterns and their applications to Internet-of-Vehicles: A comprehensive survey. Sci. China Inf. Sci..

[41] Yang H., Xiong Z., Zhao J., Niyato D., Xiao L., Wu Q. (2020). Deep reinforcement learning-based intelligent reflecting surface for secure wireless communications. IEEE Trans. Wirel. Commun..

[42] Ye H., Li G.Y., Juang B.H.F. (2019). Deep reinforcement learning based resource allocation for V2V communications. IEEE Trans. Veh. Technol..

[43] François-Lavet V., Henderson P., Islam R., Bellemare M.G., Pineau J. (2018). An introduction to deep reinforcement learning. Found. Trends® Mach. Learn..

[44] Mnih V., Badia A.P., Mirza M., Graves A., Lillicrap T., Harley T., Silver D., Kavukcuoglu K. Asynchronous methods for deep reinforcement learning. Proceedings of the 33rd International Conference on Machine Learning (PMLR).

[45] Bahdanau D., Brakel P., Xu K., Goyal A., Lowe R., Pineau J., Courville A., Bengio Y. (2016). An actor-critic algorithm for sequence prediction. arXiv.

[46] Pitis S. Rethinking the discount factor in reinforcement learning: A decision theoretic approach. Proceedings of the AAAI Conference on Artificial Intelligence.

[47] Amit R., Meir R., Ciosek K. Discount factor as a regularizer in reinforcement learning. Proceedings of the 37th International Conference on Machine Learning (PMLR).

[48] Hinton G., Srivastava N., Swersky K. (2012). Neural networks for machine learning lecture 6a overview of mini-batch gradient descent. Cited on.

[49] Xu J., Ai B., Quek T.Q., Liuc Y. Deep reinforcement learning for interference suppression in RIS-aided high-speed railway networks. Proceedings of the 2022 IEEE International Conference on Communications Workshops (ICC Workshops).

[50] Zhu Y., Li M., Liu Y., Liu Q., Chang Z., Hu Y. DRL-based joint beamforming and BS-RIS-UE association design for RIS-assisted mmWave networks. Proceedings of the 2022 IEEE Wireless Communications and Networking Conference (WCNC).

[51] Mei H., Yang K., Liu Q., Wang K. (2022). 3D-trajectory and phase-shift design for RIS-assisted UAV systems using deep reinforcement learning. IEEE Trans. Veh. Technol..

[52] Zhang S., Zhang R. On the capacity of intelligent reflecting surface aided MIMO communication. Proceedings of the 2020 IEEE International Symposium on Information Theory (ISIT).

